# Aesthetic Preferences for Replacement of Missing Maxillary Lateral Incisors: A Comparison of Canine Substitution, Implants and Resin‐Bonded Bridges Among Australian Dental Professionals and Laypeople

**DOI:** 10.1111/adj.13080

**Published:** 2025-06-03

**Authors:** Jason Guo, John M. Razza, Richard J. H. Lee, Steven Naoum, Mithran S. Goonewardene

**Affiliations:** ^1^ UWA Dental School The University of Western Australia Perth Australia

**Keywords:** canine substitution, dental aesthetics, dental implants, missing lateral incisors, orthodontic space closure, prosthetic replacement, resin‐bonded bridges

## Abstract

**Background:**

Aesthetic outcomes of treatment options for missing maxillary lateral incisors significantly influence patient satisfaction. This study compared aesthetic treatment outcome preferences for canine substitution, resin‐bonded bridges and implants among Australian orthodontists, prosthodontists, general dentists and laypeople.

**Methods:**

A cross‐sectional online survey assessed attractiveness ratings and preferences using a ranking system and a Likert scale. One‐way and two‐way ANOVA examined differences by respondent group and treatment type.

**Results:**

Among 547 respondents (orthodontists: 117, prosthodontists: 56, general dentists: 167, laypeople: 207), significant differences were found in aesthetic ratings (*p* < 0.001). Canine substitution was rated most attractive (mean rank = 1.43), followed by resin‐bonded bridges (1.78), while implants (2.81) and no treatment (3.99) were rated lower. Two‐way ANOVA showed significant main effects of treatment type and respondent group (*p* < 0.001), with an interaction effect indicating varied preferences among groups.

**Conclusions:**

Canine substitution was the preferred treatment outcome aesthetically, particularly among orthodontists. Resin‐bonded bridges were also favoured. These findings highlight the impact of professional background on aesthetic preferences and emphasise the need for patient‐centred treatment planning.

**Summary**

- This clinical relevance of this study lies in its potential to guide evidence‐based treatment planning for missing maxillary lateral incisors by integrating aesthetic preferences into clinical decision‐making.
- Dental agenesis has a psychological impact on patients; therefore, understanding how different treatment modalities are perceived by specialists, general dentists and laypeople is important for improving patient outcomes.
- By addressing gaps in the knowledge regarding aesthetic outcomes in the Australian population, this study may inform clinicians on treatment preferences, aiding in personalised care.
- Furthermore, highlighting subjective variations in aesthetic assessment reinforces the need for shared decision‐making between patients and dental professionals to achieve optimal aesthetic results.

## 1 Introduction

Aesthetic concerns have emerged as a predominant driver for patients seeking treatment in contemporary dental practice [1]. Dental agenesis is reported to affect 6.3% of the Australian population and is characterised by the frequent absence of maxillary lateral incisors, the second most commonly missing teeth excluding third molars [2, 3]. The absence of these teeth can lead to significant psychological distress, primarily due to compromised aesthetics, which can adversely affect an individual's self‐esteem [4, 5, 6]. Consequently, dental practitioners must consider aesthetics as part of the options for replacement of the maxillary lateral incisors.

Three principal treatment strategies for missing maxillary lateral incisors include no treatment, cosmetic canine recontouring, and resin bonding following orthodontic space closure [7, 8, 9] and space opening for prosthetic replacement, involving implants, traditional or resin‐bonded bridges [4, 7, 10]. The clinician is challenged with selecting the optimal treatment option with careful consideration of multiple factors, including functional outcomes, skeletal and dental relationships, periodontal health, financial cost, potential biological impact and aesthetic results [8, 10, 11, 12]. Despite the importance of aesthetics in treatment planning, there remains no clear consensus on which option provides superior aesthetic outcomes. Previous research indicates that aesthetic assessments can be highly subjective, with significant variations observed based on the assessor's professional background [13, 14, 15]. Moreover, factors influencing smile attractiveness extend beyond professional judgement, encompassing personal characteristics such as sex, age and income [13], as well as intraoral features like the ideal width‐to‐height ratio of maxillary anterior teeth [16, 17], tooth alignment [18] and gingival contour [19].

The assessment of aesthetic outcomes, particularly in the context of anterior tooth replacement, is fraught with variability. Armbruster et al. [20] cautioned that including aesthetics as a factor in evaluating treatment results might be misleading for patients, given the inherent subjectivity of aesthetic judgement, which can vary not only between individuals but also among dental professionals. Pini et al. [21] found marked differences in smile perceptions among professionals, orthodontic patients and laypeople, with dentists tending to be more critical of dental appearance. This critical perspective may stem from professionals' preference for idealised outcomes, considering factors that may be less relevant to laypeople [15]. For example, patients may be more tolerant of smile deviations, such as midline shifts or variations in the long axes of the lateral incisors, compared to dentists and orthodontists [13].

The aesthetic appeal of orthodontic space closure with canine substitution versus space opening with prosthetic replacement has been explored, with Qadri et al. [22] finding that laypeople generally perceive space closure as more attractive when replacing missing maxillary lateral incisors. Additionally, patients treated with orthodontic space closure and teeth recontouring have reported higher satisfaction with their dental appearance compared to those who underwent prosthetic replacement [4, 21]. However, there is currently no evidence on the aesthetic outcomes of different treatment options for missing maxillary lateral incisors within the Australian population, nor how these outcomes are judged across Australian specialist dentists, general dentists and laypeople.

Although it is acknowledged that there are significant variations in the complexity of various restorative procedures, the aim of the present study is to simply establish the aesthetic preference of Australian orthodontists, prosthodontists, general dentists and laypeople when replacing missing maxillary lateral incisors.

## 2 Material and Methods

All participants provided informed consent prior to their inclusion in the study, and the study protocol was approved by the University of Western Australia Human Research Ethics Committee (Project Identifier No. 2023/ET000743), ensuring that participants' rights were protected throughout the study.

**FIGURE 1:**
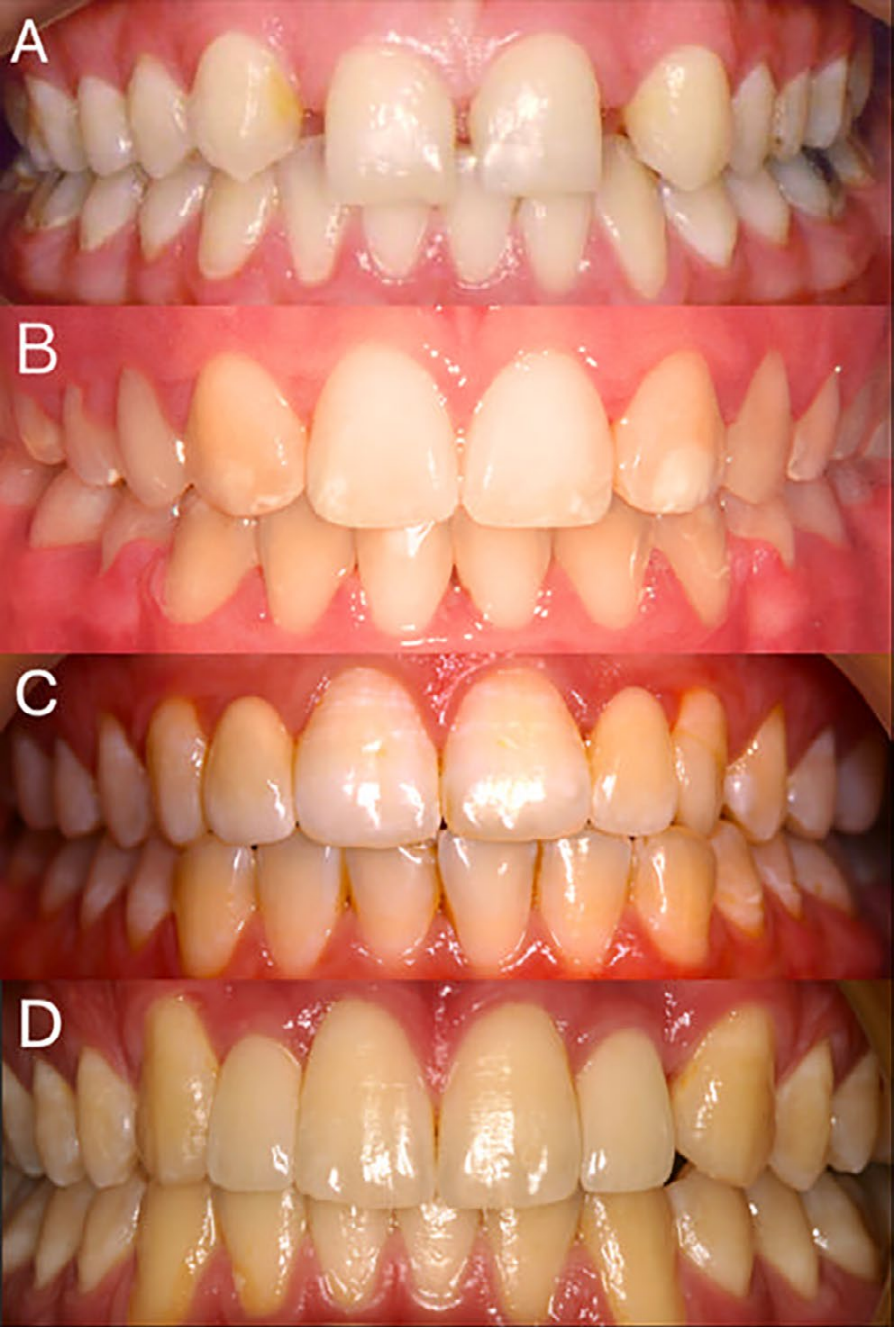
Representative sample images as presented in part one and part two of the survey. (A) No treatment. (B) Canine substitution. (C) Resin‐bonded bridges. (D) Implant supported restorations.

A cross‐sectional study design was used with data collected through an online survey tool, Qualtrics XM (https://www.qualtrics.com/en‐au/). The survey aimed to evaluate the aesthetic appeal of various treatment options by employing a numerical rating scale to assess post‐treatment images, with untreated images included as controls. Treatment options were orthodontic space closure with canine substitution, implants or resin‐bonded bridges. The images in the survey were sourced from Australian dentists, prosthodontists and orthodontists. Criteria for the images were: bilateral or unilateral absence of maxillary lateral incisors, no other missing teeth in the same quadrant except the lateral incisors, intraoral frontal view and unrestored anterior teeth aside from the prosthetic replacement or canine substitution of the lateral incisor. Images were standardised with cropping to exclude the lips and to retain the full extent of the attached gingiva and teeth (Figure 1).

**FIGURE 2:**
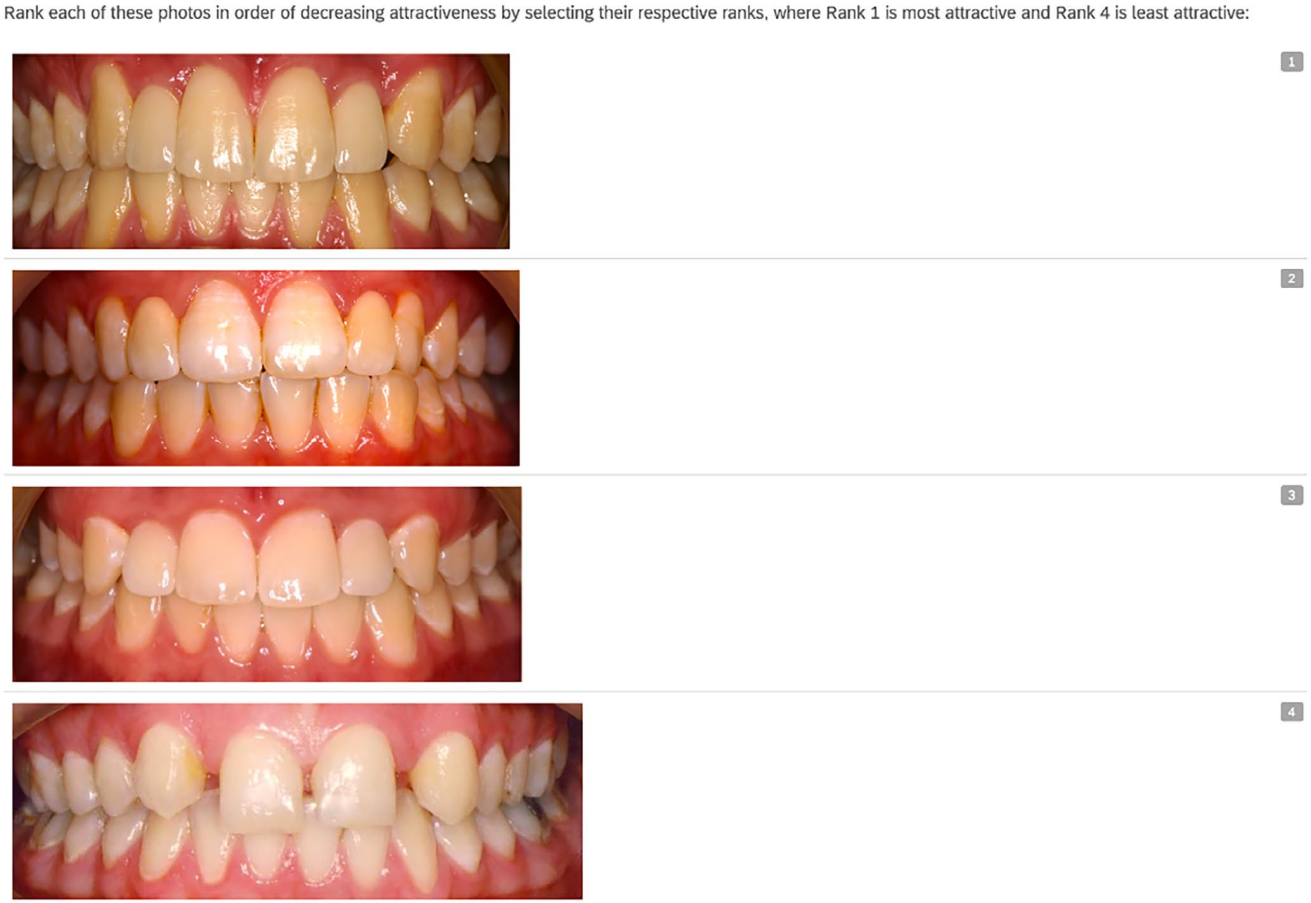
Part one of the survey where participants rank each image from most attractive (rank 1) to least attractive (rank 4).

**FIGURE 3:**
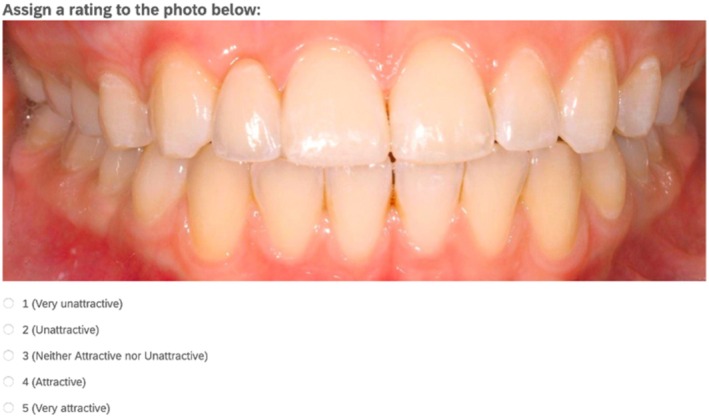
Example of a question from part two of the survey where participants rate each image on a 5‐point scale from 1 (very unattractive) to 5 (very attractive).

The survey consisted of three parts after assessing if the survey participant was an orthodontist, prosthodontist, general dentist or layperson. The complete set of survey questions is presented in Table 1. Part one involved ranking a set of four images from most to least aesthetic (Figure 2). Each image represented a different treatment option and a control image with no treatment for missing maxillary lateral incisors. The images were ranked on a scale with the most attractive image being ranked 1 and the least attractive ranked 4. The frequency of each score was analysed to determine which treatment option was perceived as most aesthetic on average compared to others. In part two of the survey, participants evaluated the aesthetic appeal of the treatment options for replacing missing lateral incisors, alongside control images showing no treatment (Figure 3). Each image was rated independently on a five‐point Likert scale ranging from ‘Very Unattractive’ (1) to ‘Very Attractive’ (5). To prevent bias, participants were not informed about the treatment options represented in the images. A total of 16 images—four for each treatment option, including the control—were displayed in random order. Part three of the survey focused on assessing treatment preferences among Australian specialist dentists and general dentists. Dental professional participants were asked about their preferred treatment option for missing maxillary lateral incisors. The correlation between participants' treatment preferences and their aesthetic rankings of each option was analysed.

**TABLE 1:** Survey questionnaire.

Question set	
Consent	I have read the participant information provided and consent to participate in this survey Yes No
Occupational background	I am a… General Dentist Specialist Orthodontist Specialist Prosthodontist Layperson (no professional dental experience)
Ranking images from most to least aesthetic	Rank each of these photos in order of decreasing attractiveness by selecting their respective ranks, where Rank 1 is most attractive, and Rank 4 is least attractive [Image of control case] [Image of canine substitution case] [Image of resin‐bonded bridge case] [Image of implant restoration case]
Aesthetic rating of images	Assign each image a rating of attractiveness of the patient's teeth from 1 to 5 where 1 is very unattractive, and 5 is very attractive All images were randomised for each rater [Four images of control cases] [Four images of canine substitution cases] [Four images of resin‐bonded bridge cases] [Four images of implant restoration cases]
Assessment of treatment preferences among dental professionals	This question is only displayed if the occupational background was selected to be a general dentist, specialist orthodontist, or specialist prosthodontist Which treatment option would you most prefer for patients in the treatment of congenitally missing upper lateral incisors? No preference Canine substitution Resin‐bonded bridges Implant supported restorations

The sample size calculation was based on detecting a clinically meaningful difference of one point on the Likert scale between any two groups. With a conservative estimate of a standard deviation of one, 17 individuals per group (51 in total) were required to achieve 80% power and a Type I error probability of 0.05. Participants were recruited via a combination of convenience sampling and invitations to participate through the group's respective professional societies. Orthodontists registered with the Australian Society of Orthodontists, prosthodontists registered with the Australian Prosthodontic Society, and general dentists with the Australian Dental Association were invited to participate. Laypeople were recruited in person at public forums with inclusion criteria of being 18 years of age or older, having no dental training or association, and not being immediately related to someone in the dental field.

### 2.1 Statistical Analysis

The primary analysis involved a one‐way ANOVA to evaluate differences in attractiveness ratings across participant groups (orthodontists, prosthodontists, general dentists and laypeople). The response variable was the attractiveness rating for each treatment option, and group affiliation served as the fixed effect. A significance level of 0.05 was used for all statistical tests. To analyse aesthetic rankings of treatment options, rankings were collated to determine overall attractiveness scores for each treatment type. A two‐way ANOVA was conducted with treatment type and participant group as fixed effects. The response variable was the attractiveness rating, and interaction effects between group affiliation and treatment type were assessed. A significance level of 0.05 was applied. Additionally, Spearman correlation analysis was performed to explore relationships between participants' aesthetic rankings and their preferred treatment choices. Correlation coefficients were calculated for each treatment option to determine whether a significant relationship existed between perceived attractiveness and treatment preference. All statistical analyses were conducted using R software [23], and results were interpreted with a focus on clinical and aesthetic implications for the management of missing lateral incisors.

## 3 Results

A total of 547 valid responses were received for the survey. Of these, 117 were orthodontists, 56 were prosthodontists, 167 were general dentists and 207 were laypeople.

An analysis of the mean ranks for the attractiveness of different treatment options was conducted based on responses from the total (*n* = 547) participants. The mean ranks, standard deviations and standard errors of the mean (SEM) for each treatment are presented in Table 2. Canine substitution was rated as the most attractive with the lowest mean rank of 1.430 (SD = 0.632, SEM = 0.027), followed by resin‐bonded bridges with a mean rank of 1.779 (SD = 0.596, SEM = 0.026). Implants had a higher mean rank of 2.806 (SD = 0.516, SEM = 0.022), indicating they were less preferred compared to canine substitution and resin‐bonded bridges. The control group was the least attractive with the highest mean rank of 3.985 (SD = 0.120, SEM = 0.005).

**TABLE 2:** Summary of mean rankings for attractiveness of treatment options.

Group	*n*	Canine substitution			Implants			Resin‐bonded bridges			No treatment		
		M	SD	SEM	M	SD	SEM	M	SD	SEM	M	SD	SEM
Orthodontist	117	1.513	0.715	0.066	2.821	0.385	0.036	1.667	0.572	0.053	4.000	0.000	0.000
Prosthodontist	56	1.750	0.837	0.112	2.750	0.437	0.058	1.500	0.505	0.067	4.000	0.000	0.000
General dentist	167	1.473	0.638	0.049	2.629	0.772	0.060	1.946	0.688	0.053	3.952	0.214	0.016
Layperson	207	1.261	0.440	0.031	2.957	0.204	0.014	1.783	0.508	0.035	4.000	0.000	0.000
Total	547	1.430	0.632	0.027	2.806	0.516	0.022	1.779	0.596	0.026	3.985	0.120	0.005

Abbreviations: M, mean; *n*, sample size; SD, standard deviation; SEM, standard error of the mean.

Comparisons were made among orthodontists, prosthodontists, general dentists and laypeople. A one‐way ANOVA was conducted (Table 3) to compare the effect of occupational group on the attractiveness scores of the treatment options as well as for no treatment (control). The analysis revealed a statistically significant effect of group affiliation across all treatment categories. Implant treatment received significantly different ratings among the four groups (*F*_3,2188_ = 78.18, *p* < 0.001). Resin‐bonded bridge ratings also varied significantly between the groups (*F*_3,2188_ = 78.77, *p* < 0.001). Canine substitution demonstrated the largest variation in ratings (*F*_3,2188_ = 76.57, *p* < 0.001). For the control photographs, there was a significant difference in aesthetic ratings between the groups, albeit with a lower *F*‐statistic (*F*_3,2188_ = 17.46, *p* < 0.001). These results indicate the perceived attractiveness of treatment options for missing lateral incisors is significantly influenced by the rater's professional background or lay status, suggesting differing aesthetic preferences and professional biases among orthodontists, prosthodontists, general dentists and laypeople.

**TABLE 3:** Results of one‐way ANOVA by treatment options.

Source	SS	df	MS	*F*	*p*
Canine substitution	175.88	3	58.63	76.57	< 0.0001
Implants	183.14	3	61.05	78.18	< 0.0001
Resin‐bonded bridges	171.99	3	57.33	78.77	< 0.0001
No treatment	17.62	3	5.87	17.46	< 0.0001

Abbreviations: df, degrees of freedom; *F*, *F*‐statistic; MS, mean square; SS, sum of squares.

A two‐way ANOVA (Table 4) used data from part 2 of the survey to assess the attractiveness of the treatment options across the subject groups, with treatment type and group affiliation as fixed factors. There was a significant effect of the rater group on attractiveness ratings (*F*_3,8736_ = 165.71, *p* < 0.001), indicating that the perceived attractiveness of treatment options varied significantly between the affiliated groups. The type of treatment significantly influenced attractiveness ratings (*F*_3,8736_ = 2741.58, *p* < 0.001), which demonstrates that the aesthetics of the treatment options differed significantly. A significant interaction effect was observed between the rater groups and treatment type (*F*_9,8736_ = 3816, *p* < 0.001), which suggests that the effect of treatment type on attractiveness ratings varied depending on the rater group. In other words, the relative attractiveness of each treatment option was perceived differently by the different occupational groups.

**TABLE 4:** Results of two‐way ANOVA (treatment type × group affiliation).

Source	SS	df	MS	*F*	*p*
Treatment type	5368.15	3	1789.38	2741.58	< 0.0001
Group affiliation	328.47	3	108.16	165.71	< 0.0001
Interaction (treatment type × group affiliation)	224.15	9	24.91	38.16	< 0.0001
Residual	5701.84	8736	0.65		

Abbreviations: df, degrees of freedom; *F*, *F*‐statistic; MS, mean square; SS, sum of squares.

There was a statistically significant effect of treatment type on attractiveness scores, *F*_3,2172_ = 2952.64, *p* < 0.001, indicating attractiveness scores differed significantly between the treatment options. Conversely, the main effect of rater group was not significant, *F*_3,2172_ < 0.001, *p* = 1.000, suggesting that overall respondent groups did not differ significantly in their attractiveness ratings. The interaction between treatment type and group affiliation was statistically significant, *F*_9,2172_ = 15.28, *p* < 0.001. This finding indicates that the differences in attractiveness scores between treatment options were influenced by the group affiliation, suggesting variability in how subject groups perceived the relative aesthetic appeal of the treatment options.

A Spearman correlation analysis (Table 5) was conducted to evaluate the relationship between the aesthetic rankings of treatment options and their selection as the preferred treatment by dental professionals. For canine substitution, the correlation between aesthetic rankings and preference for canine substitution was negligible (Spearman *r*_*s*_ = −0.044, *p* = 0.417), indicating no statistically significant relationship. For implant treatment, a weak positive correlation was observed (*r*_*s*_ = 0.019, *p* = 0.720), indicating no significant relationship between aesthetic ranking and treatment preference. Similarly, the correlation for resin‐bonded bridges was very weak and not statistically significant (*r*_*s*_ = 0.019, *p* = 0.728).

**TABLE 5:** Spearman correlation analysis between rankings and treatment preferences.

	Spearman correlation	*p*
Canine substitution	−0.044	0.417
Implants	0.019	0.720
Resin‐bonded bridges	0.019	0.728
No treatment	0.017	0.750

Treatment preferences among orthodontists, prosthodontists and general dentists showed distinct patterns (Table 6). Orthodontists predominantly favoured canine substitution, with 79.49% selecting it as their preferred treatment for missing lateral incisors. A smaller proportion (17.95%) expressed no preference, while only 2.56% opted for resin‐bonded bridges, and none selected implants. Prosthodontists displayed a more varied distribution of preferences. Half (50%) reported no preference, while 25% favoured resin‐bonded bridges, and 12.5% preferred implants, indicating a preference for fixed restorative solutions. A minority (12.5%) chose canine substitution, and none selected implants. General dentists exhibited a more evenly distributed set of preferences. Implants were the most selected treatment option (34.13%), followed by no preference (24.55%), resin‐bonded bridges (22.75%) and canine substitution (18.56%).

**TABLE 6:** Summary of treatment preferences by group affiliation.

Group	*n*	Canine substitution *n*, %	Implants *n*, %	Resin‐bonded bridges *n*, %	No preference *n*, %
Orthodontists	117	93 (79.49%)	0 (0.0%)	3 (2.56%)	21 (17.95%)
Prosthodontists	56	5 (8.93%)	11 (19.64%)	12 (21.43%)	28 (50.0%)
General dentists	167	31 (18.56%)	57 (34.13%)	38 (22.75%)	41 (24.55%)

Abbreviation: *n*, sample size.

## 4 Discussion

This study evaluated the aesthetic preferences for different treatment options for missing maxillary lateral incisors among orthodontists, prosthodontists, general dentists and laypeople. The results demonstrated significant differences in attractiveness ratings and treatment preferences from an aesthetic outcome perspective across the groups.

The ANOVA analyses revealed significant differences in aesthetic ratings across respondent groups, confirming that professional background influences aesthetic judgements. Dental professionals, including orthodontists, prosthodontists and general dentists, tend to have more critical and idealised views of aesthetics compared to laypeople, which is consistent with previous research [14, 15, 21, 24, 25, 26]. Additionally, the two‐way ANOVA showed that both group affiliation and treatment type significantly affected attractiveness ratings, with an interaction between the two. This interaction suggests that raters' professional or non‐professional backgrounds shape their perceptions of treatment options, likely due to varying levels of clinical experience. These results highlight the importance of acknowledging the subjectivity of aesthetics in treatment planning.

The analysis of the mean ranks for attractiveness showed that canine substitution was rated as the most attractive option aesthetically, followed by resin‐bonded bridges and implants, with the control group rated the least attractive. This preference for canine substitution is consistent with previous research indicating that orthodontic space closure and recontouring are often viewed more favourably by both patients and clinicians from an aesthetic view point [27, 28, 29]. While implants are a common choice for tooth replacement, the lower rankings in terms of attractiveness may reflect concerns about implant visibility or gingival contour, factors that influence overall smile aesthetics [30, 31]. The control group's low ranking further emphasises the strong preference for active treatment among respondents.

The frequency analysis of preferred treatment options across professional groups highlighted further differences in aesthetic preferences. General dentists favoured implants, while specialist dentists such as orthodontists overwhelmingly preferred canine substitution. This disparity likely reflects differences in training, clinical experience and priorities in managing missing maxillary lateral incisors [4, 8, 9, 10, 32]. Orthodontists' strong preference for canine substitution (79.49%) compared to implants or resin‐bonded bridges is consistent with their specialised expertise in orthodontic space closure. This may be due to expectations for increased stability and long‐term aesthetic results compared to prosthetic replacements [9, 32, 33].

Several studies have shown that orthodontic space closure, combined with recontouring of canines, provides a natural‐looking aesthetic outcome, especially in younger patients where long‐term implant viability may be compromised by continuing growth and bone development [4, 8, 9]. General dentists, on the other hand, demonstrated a more varied preference, with a significant portion (34.13%) favouring implants. This inclination toward implant‐based solutions may reflect the general dentist's broader involvement in restorative treatments and the increasing accessibility and success rates of dental implants over recent decades [34, 35].

Implants offer a prosthetic solution that avoids reliance on adjacent teeth, which may appeal to general dentists seeking to preserve tooth structure and offer long‐lasting results with minimal orthodontic intervention. Additionally, general dentists may not have the same level of confidence or clinical experience in orthodontic space closure and therefore view implants as a more straightforward and predictable option [32, 36]. Prosthodontists presented a more balanced distribution in treatment preferences, with some favouring resin‐bonded bridges (21.43%) and others preferring implants (19.64%). This reflects their focus on prosthetic replacement and restoration of missing teeth, where both implants and resin‐bonded bridges offer viable aesthetic solutions. Prosthodontists are often tasked with providing restorative solutions for patients who have either already completed orthodontic treatment or require a prosthetic replacement due to patient preference or other factors such as periodontal health or bone availability for implants. Their training allows them to weigh both functional and aesthetic outcomes of prosthetic interventions, potentially explaining the more even distribution of preferences compared to the orthodontists and general dentists. These findings echo the variability in professional judgement noted by Pini et al. [21].

These differences underscore the importance of individualised treatment planning, where patient preferences, dental health and professional recommendations must be balanced. For example, while orthodontists may favour space closure for younger patients due to its predictability, implants might be a more suitable choice for adults with fully developed skeletal structures who seek a less time‐costly solution. Moreover, the patient's overall oral health, such as periodontal status and occlusion, can influence the treatment planning process.

This study contributes to the existing literature by providing insights into the aesthetic preferences of Australian dental professionals and laypeople regarding the replacement of missing maxillary lateral incisors. While previous research has explored aesthetic outcomes in other populations for missing maxillary lateral incisors [7, 15, 20, 22, 24], this study is the first to examine these preferences within the Australian context. The significant differences observed between orthodontists, prosthodontists, general dentists and laypeople highlight the importance of individualised treatment planning.

Moreover, the strong preference for canine substitution among specialist dentists suggests that this treatment may be favoured for its aesthetic outcomes, potentially informing future treatment guidelines and patient consultations. These findings also provide data for dental professionals in balancing aesthetic, functional and patient‐centred care when planning treatments for missing maxillary lateral incisors.

### 4.1 Limitations

The survey relied on subjective evaluations of attractiveness, which are inherently subjective and variable. Although this variability was accounted for by including multiple groups of respondents, future studies could benefit from a larger and more diverse sample size to further validate these findings. Additionally, the use of photographic images for the survey without comprehensive dynamic smile analysis may not fully capture the three‐dimensional aesthetics of different treatment options [37]. A combination of photographs and intraoral scans could provide a more comprehensive evaluation. Moreover, this study did not explore the complexities of clinical decision making, including the relative risks and benefits of active orthodontic treatment and the various restorative dentistry approaches following space closure or the issues related to prosthetic replacement. Finally, the study did not explore the influence of demographic factors such as age, gender or socioeconomic status, which have been shown to affect aesthetic perceptions in other studies [38, 39].

### 4.2 Conclusions

The findings of this study indicate that both treatment type and occupational background significantly influence perceptions of attractiveness for missing maxillary lateral incisors. Canine substitution was generally rated as the most attractive option, particularly among orthodontists. These results suggest that treatment decisions based on aesthetics for maxillary lateral incisor replacement should carefully consider both professional recommendations and patient preferences. Future research should explore the long‐term aesthetic and functional outcomes of different treatment approaches and how these align with patient satisfaction.

## Author Contributions

**Jason Guo:** conceptualization, methodology, software, data curation, investigation, formal analysis, visualization, writing – original draft. **John M. Razza:** conceptualization, methodology, writing – review and editing. **Richard J. H. Lee:** conceptualization, methodology, writing – review and editing. **Steven Naoum:** conceptualization, methodology, writing – review and editing. **Mithran S. Goonewardene:** conceptualization, methodology, supervision, resources, project administration, funding acquisition, writing – review and editing.

## Conflicts of Interest

The authors declare no conflicts of interest.
